# Identification of RNA-splicing factor Lsm12 as a novel tumor-associated gene and a potent biomarker in Oral Squamous Cell Carcinoma (OSCC)

**DOI:** 10.1186/s13046-022-02355-9

**Published:** 2022-04-21

**Authors:** Yan Dong, Liyan Xue, Yan Zhang, Caiyun Liu, Yanguang Zhang, Na Jiang, Xiaoyan Ma, Fangyu Chen, Lingxia Li, Liyuan Yu, Xuefeng Liu, Shujuan Shao, Shufang Guan, Jian Zhang, Qingchun Xiao, Hui Li, Ailing Dong, Lijie Huang, Chenyang Shi, Yan Wang, Ming Fu, Ning Lv, Qimin Zhan

**Affiliations:** 1 College of Stomatology, Dalian Medical University, Dalian, 116044 Liaoning China; 2 Department of Pathology, National Cancer Center/National Clinical Research Center for Cancer/Cancer Hospital, Chinese Academy of Medical Sciences and Peking Union Medical College, Beijing, 100021 China; 3 Key Laboratory of Carcinogenesis and Translational Research (Ministry of Education/Beijing), Laboratory of Molecular Oncology, Peking University Cancer Hospital & Institute, Beijing, 100142 China; 4 Hospital of Stomatology, Dalian Medical University, Dalian, 116027 Liaoning China; 5 Institute of Cancer Stem Cell, Dalian Medical University, Dalian, 116044 Liaoning China; 6 Liaoning Key Laboratory of Proteomics, Dalian Medical University, Dalian, 116044 Liaoning China; 7 State Key Laboratory of Molecular Oncology, National Cancer Center/National Clinical Research Center for Cancer/Cancer Hospital, Chinese Academy of Medical Sciences and Peking Union Medical College, Beijing, 100021 China

**Keywords:** OSCC, Lsm12, Alternative splicing, USO1 exon 15, Novel biomarker

## Abstract

**Background:**

Oral squamous cell carcinoma (OSCC) is one of the common cancers worldwide. The lack of specific biomarkers and therapeutic targets leads to delayed diagnosis and hence the poor prognosis of OSCC patients. Thus, it is urgent to identify effective biomarkers and therapeutic targets for OSCC.

**Methods:**

We established the golden hamster carcinogenic model of OSCC induced by 7,12-dimethylbenz(a) anthrancene (DMBA) and used mRNA microarrays to detect the differentially expressed genes (DEGs). DEGs were validated in OSCC clinical tissue microarrays using immunohistochemistry method. Whole transcriptome sequencing was performed to obtain an overview of biological functions of Lsm12. PCR assay and sequencing were employed to investigate the alternative splicing of genes regulated by Lsm12. Cell proliferation, colony formation, Transwell migration and invasion assay and in vivo tumor formation assay were performed to investigate the roles of Lsm12 and two transcript variants of USO1 in OSCC cells.

**Results:**

Lsm12 was identified to be significantly up-regulated in the animal model of OSCC tumorigenesis, which was validated in the clinical OSCC samples. In the paired normal tissues, Lsm12 staining was negative (91%, 92/101) or weak, while in OSCC tissues, positive rate is 100% and strong staining spread over the whole tissues in 93 (93/101, 92%) cases. Lsm12 overexpression significantly promoted OSCC cell growth, colony formation, migration and invasion abilities, while Lsm12 knockdown showed the opposite trends on these phenotypes and obviously inhibited the tumor formation in vivo. Furthermore, Lsm12 overexpression caused the inclusion of USO1 exon 15 and Lsm12 knockdown induced exon 15 skipping. Exon 15-retained USO1 significantly promoted the malignant phenotypes of OSCC cells when compared with the exon 15-deleted USO1.

**Conclusions:**

We identified Lsm12, a novel tumorigenesis-related gene, as an important regulator involved in OSCC tumorigenesis. Lsm12 is a novel RNA-splicing related gene and can regulate the alternative splicing of USO1 exon 15 which was associated closely with OSCC carcinogenesis. Our findings thus provide that Lsm12 might be a potent biomarker and potential therapeutic target for OSCC.

**Supplementary Information:**

The online version contains supplementary material available at 10.1186/s13046-022-02355-9.

## Background

Oral cancer is common worldwide with an estimated over 300,000 new cases and 150,000 deaths annually [1]. Oral squamous cell carcinoma (OSCC) accounts for the vast majority of oral cancers [2, 3]. A lack of early diagnostic biomarkers and therapeutic targets leads to a delayed clinical diagnosis and poor prognosis of OSCC. Therefore, it is urgent to identify early molecular markers for OSCC and to explore the underlying mechanisms of OSCC tumorigenesis.

Many studies were carried out to explore the biomarkers and mechanism of OSCC tumorigenesis and a large number of tumor-related genes have been identified [4–11]. For example, Zinc-finger protein 750 (ZNF750) was reported to arrest the cell cycle of OSCC cells in G0/G1 phase [5]. AUNIP knockdown suppressed OSCC cell growth and resulted in G0/G1 phase arrest in OSCC cells [7]. Using the 7,12-dimethylbenz(a) anthrancene (DMBA)-induced golden hamster model, an optimal model for oral oncogenesis, the carcinogenesis mechanism and treatment of OSCC were studied [12–18]. Administration of nimbolide to golden hamsters painted with DMBA for 12 weeks led to significantly delayed tumor growth and reduced tumor burden. Nimbolide inhibits cytoprotective autophagy to activate apoptosis by modulating the PI3K/Akt/GSK-3β signaling pathway in oral cancer [12]. Hesperetin showed a potential effect on DMBA-induced oral carcinogenesis because hesperetin treatment could reverse the expression of apoptotic and proliferative markers to near normal in buccal mucosal tissues of golden hamsters [17]. However, robust markers and therapeutic targets of OSCC that can be used in the clinical setting have not yet been identified.

Cancer is usually considered to be caused by the aberrant expression of tumor-related genes [19, 20]. High-throughput analysis of cancer-associated gene expression profile by using mRNA microarray is effective in identifying potential biomarkers and therapeutic targets [21, 22]. Therefore, in the present study, we established the golden hamster model of OSCC tumorigenesis and used mRNA microarrays to carried out the high-throughput analysis of differentially expressed genes (DEGs) among the normal, hyperplasia, mild/moderate dysplasia, papilloma and squamous cell carcinoma tissues in the model. We focused on the unknown tumorigenesis-associated and upregulated DEGs which were validated in the tissue microarrays composed of 101 cases of OSCC tissues and the paired adjacent normal tissues.

Immunohistochemistry analysis revealed that Lsm12 expression was significantly upregulated in OSCC tissues. The distribution characteristics of Lsm12 protein indicated that Lsm12 could be a potent marker and has great clinical applications. In addition, GEPIA (Gene Expression Profiling Interactive Analysis) database (http://gepia.cancer-pku.cn/detail.php) was used to find out that expression of Lsm12 in many types of human malignant tumor tissues was also significantly upregulated compared with the paired normal tissues, which indicated that Lsm12 is a very critical gene in the tumorigenesis.

**Fig. 1:**
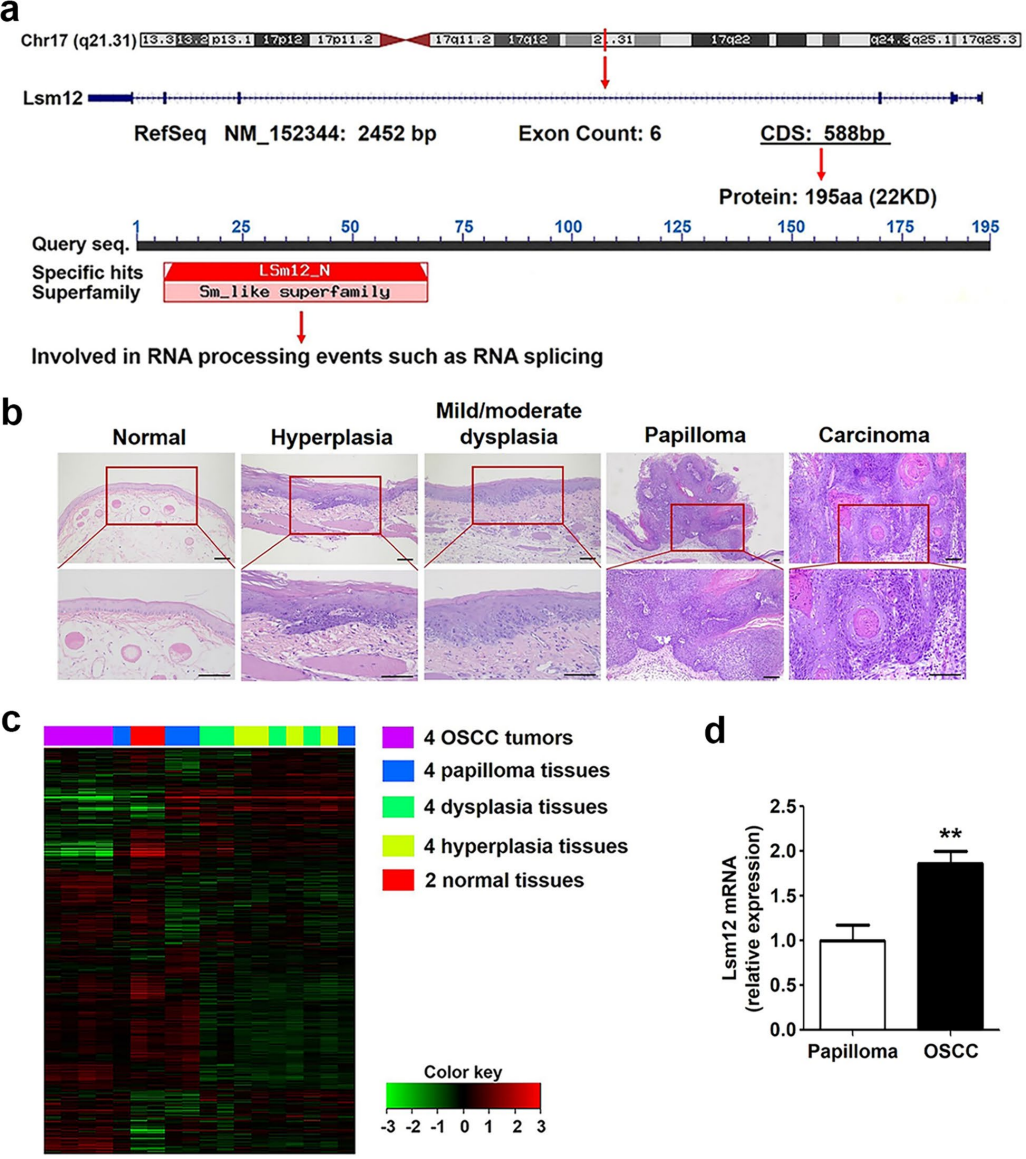
Lsm12 expression is upregulated in the model of OSCC tumorigenesis. **a** The position of Lsm12 gene on the chromosome and its transcription and translation products. Lsm12 belongs to Sm family which is involved in RNA splicing. **b** Histopathological images of normal epithelium, hyperplasia, mild/moderate dysplasia, papilloma and squamous cell carcinoma in the animal model of OSCC tumorigenesis. The papilloma is accompanied by severe dysplasia. Bar, 100 μm. **c** The clustergram of mRNA microarray showed genome-wide expression profiles in four squamous cell carcinoma tissues (purple), four papilloma tissues (dark blue), four dysplasia tissues (light green), four hyperplasia tissues (yellow) and two normal tissues (red). **d** Real time PCR results validated that Lsm12 expression at mRNA level was significantly upregulated in OSCC tissues compared with that in papilloma tissues (*P* < 0.01), which were consistent with the results of mRNA microarray data analysis

UCSC genome browser database (http://genome-asia.ucsc.edu/cgi-bin) and NCBI database (https://www.ncbi.nlm.nih.gov/) show that Lsm12 has five transcript variants among which Lsm12 transcript variant 3 (NCBI Reference Sequence: NM_152344) is first discovered (Fig. 1a). The present study was conducted to explore the roles and mechanism of Lsm12 transcript variant 3 in OSCC tumorigenesis.

According to the NCBI database, Lsm12 protein contains a like-Sm protein domain (7-67aa), which indicates that Lsm12 belongs to a family of like-Sm proteins involved in RNA processing events such as pre-mRNA splicing. Many alternative splicing (AS) changes contribute to the tumorigenesis [23–26]. However, the biological function of Lsm12 in the tumorigenesis is still unclear. Yao R et al. reported that in the Saccharomyces cerevisiae, overexpression of Lsm12 enhanced cell growth while deletion of Lsm12 caused growth defects under oxidative stress [27].

In the present study, we discovered that Lsm12 plays key roles in the tumorigenesis of OSCC and can modulate alternative splicing of USO1 gene. USO1 has been identified as a tumor-related gene [28–31]. But the underlying molecular mechanism of USO1 gene in the tumorigenesis remains unclear. In this study, we explored the role of alternative splicing of USO1 in the tumorigenesis.

## Methods and materials

### Cell culture

Two OSCC cell lines, human SCC-25 and CAL 27 were originally obtained from American Type Culture Collection (ATCC, USA). SCC-25 cells were cultured in 1:1 mixture of DMEM and Ham’s F12 medium supplemented with 500 ng/ml hydrocortisone and 20% fetal bovine serum. CAL 27 cells were cultured in DMEM medium supplemented with 20% fetal bovine serum. All cells were grown in a humidified 5% CO_2_ incubator at 37 °C.

### Induction of OSCC tumors

40 Syrian golden hamsters (90-110 g in weight, half male and half female) obtained from Beijing Vital River Laboratory Animal Technology (China) were used in this study. Animals were randomly divided into four experimental groups, including group A (*n* = 8), B (*n* = 8), C (*n* = 8), D (*n* = 10), and one control group (*n* = 6). In four experimental groups, left cheek pouches of the animals were painted three times per week with 50 μl of 0.5% DMBA (Sigma-Aldrich) solution in acetone. Group A had undergone DMBA treatment for 4 weeks, group B for 7 weeks, group C for 10 weeks and group D for 14 weeks. The control group animals were painted with 50 μl of acetone solution alone for 14 weeks. All animals were housed at 22 °C, with standard 12-h light/dark cycles. Food and water were provided ad libitum. Animals were euthanized with CO_2_.

### Histopathological diagnosis

All buccal pouches were examined and excised. For preparation of total RNA, one half of each harvested tissue was snap frozen in the liquid nitrogen and then transferred to −80 °C refrigerator to save. Another half of each tissue was immediately fixed in 10% formalin and paraffin-embedded for histopathological diagnose. Sections underwent standard hematoxylin and eosin (HE) staining. Diagnosis was done independently by two pathologists.

Hyperplasia, dysplasia, papilloma and squamous cell carcinoma were diagnosed with the established criteria [32]. Epithelial hyperplasia shows mild hyperkeratosis and prominent acanthosis, with preservation of normal cell maturation. Dysplasia is characterized by cellular atypia, loss of normal maturation and stratification, increased number of mitotic figures, increased nuclear-to-plasma ratio and loss of polarity of basal cells. Dysplasia is divided into three grades of severity. Mild dysplasia is featured by cytological atypia limited to the basal third, moderate dysplasia by extension into the middle third, and severe dysplasia by extension into the upper third. Papilloma was diagnosed by the finger-like stratified squamous epithelial projections extending from a narrow base and supported by fibrovascular cores. Papilloma can be accompanied by dysplasia. Carcinoma was diagnosed by the invasion into underlying tissues, including those originating from papilloma or from flat mucosa.

According to histopathological diagnosis, 18 corresponding frozen specimens, including 4 frozen samples from each group of hyperplasia, mild/moderate dysplasia, papilloma, squamous cell carcinoma and 2 frozen samples from the control group, were selected for mRNA microarray assay.

### mRNA microarray data Analysis

The genome wide gene expression profiles in 18 specimens were detected by Agilent rat mRNA microarray from CapitalBio Corporation (Beijing, China) and the differentially expressed genes (DEGs) among five groups, including the control, hyperplasia, mild/moderate dysplasia, papilloma and OSCC groups, were identified. The random-variance model (RVM) F-test was applied to filter the DEGs. The series test of cluster (STC) algorithm was employed to profile the gene expression time series. Pathway analysis was performed to find out the significant pathway of DEGs according to KEGG.

### Real-time PCR and semi-quantitative PCR

The purified total RNA was used for real-time PCR to detect Lsm12 expression in papilloma tissues and OSCC tissues of golden hamsters as well as stably transfected cells. Briefly, total RNA was reverse-transcribed into cDNA with a reverse transcription kit, followed by real time PCR reaction with SYBR Premix Ex Taq. The reactions were run in triplicate on a 7300 Realtime PCR system (Applied Biosystems, USA). The primer sequences used in PCR were as follows:

Lsm12 (rat) forward, TCGAACAGAAACCCCTCCTC;

reverse, TTGCATAGGCCTGGCTCAAC.

GAPDH (rat) forward, CCCATGGCAAGTTCAAAGGC;

reverse, CTTGGCTCCACCCTTCAAGT.

Lsm12 forward, CTAGAGGGCCAGCAGCTCTT;

reverse, TTCCACTTGATATGGGGGTG.

GAPDH forward, GCTGAGAACGGGAAGCTTGT;

reverse, GCCAGGGGTGCTAAGCAGTT.

To measure USO1 exon 5 and exon 15 expression, total RNA was extracted from cells and reverse-transcribed into cDNA, followed by PCR reaction. Semi-quantitative PCR was conducted on a Veriti 96-well Thermal Cycler (Applied Biosystems, USA). PCR products were electrophoresed on 3% agarose gel. The primer sequences used in PCR were as follows:

USO1 (exon 15) forward, TGCCACCCAGAAAGAACAGT;

reverse, GGGACAATTGCTTAGCCAGG.

USO1 (exon 5) forward, ATAGGTTATGCTTTGGACACACT;

reverse, GAGTGACATTTTCCTGCTGCTT.

### Tissue microarray

To detect the expression of the unknown tumorigenesis-related DEGs in OSCC patients, paraffin-embedded tissues of 101 OSCC cases were collected from the Cancer Hospital of Chinese Academy of Medical Sciences. We constructed tissue microarrays containing specimens from 101 cases of OSCC tumors and the paired adjacent normal tissues without a history of chemo-radiation therapy. Sections were stained with HE and diagnoses were confirmed by two pathologists histologically.

### Immunohistochemistry analysis

Using immunohistochemistry (IHC) method, unknown tumorigenesis-related DEGs which were successfully verified in golden hamster tissues were furtherly validated in OSCC tissue microarray. IHC was performed according to standard protocols. Tissue microarrays were incubated with rabbit anti-Lsm12 primary antibodies (Abcam, 1:400 dilution) overnight at 4 °C. After washing, the tissue microarrays were treated with horseradish peroxidase (HRP)-conjugated goat anti-rabbit IgG for 30 min. 3, 3′-Diaminobenzidine was used as the chromogen. Scores were based on the % of cells with positive staining for Lsm12 (0 = 0-5%, 1 + =6–25%, 2 + =26-50%, 3 + =51-75%, 4 + =76-100%) and the intensity of Lsm12 staining in positive areas (1 + =low intensity staining, 2 + =high intensity staining). Then final score was calculated by multiplying the percentage score and the intensity score for each case, which ranged between 0 and 8. A score of 1-4 was considered as weak and a score of 6 and 8 as strong. 0 was considered negative.

### Lentivirus production

Lsm12 shRNA sequences were designed according to the coding sequence (CDS) region of Lsm12 gene as follows,

CCGGCCCTAGCTTCACTCAATGTTACTCGAGTAACATTGAGTGAAGCTAGGGTTTTTG.

Next, Lsm12-shRNA was inserted into lentiviral vector to yield pLKO.1-Lsm12-shRNA plasmid. After DNA sequencing, pLKO.1-Lsm12-shRNA plasmid was transfected into 293 T cells and the lentiviral particles were collected at 72 h after transfection. Additionally, green fluorescent protein (GFP)-tagged lentiviral particles containing Lsm12 cDNA or USO1 transcript variant with or without exon 15, were obtained from OBiO technology (shanghai, China).

### Stable transfection

Cells were infected with lentiviral particles containing Lsm12 shRNA or Lsm12 cDNA to establish stable Lsm12 knockdown or Lsm12 overexpression cell lines. CAL 27 cells were infected with lentiviral particles containing USO1 cDNA with or without exon 15 to construct the stable cell line expressing USO1 with exon 15, named USO1-FL (full length), or expressing USO1 without exon 15, named USO1-DE15. After infection, SCC-25 and CAL 27 cells were cultured for 48 h followed by selection with 2 μg/ml and 0.8 μg/ml puromycin, respectively. Puromycin-resistant cells were selected and expanded. Lsm12 knockdown or overexpression was confirmed by real time PCR and western blotting. Expression of USO1 transcripts variants in CAL 27 cells was validated by PCR. PCR products were electrophoresed on 3% agarose gel. The bands were excised from the gel and purified. The purified products were sequenced by Genewiz, Inc. (Suzhou, China).

### Western blotting

Cells were harvested and lysed. Protein extract was quantified using QuantiPro BCA Assay Kit (Sigma) according to the manufacturer’s instructions. Identical quantities of proteins were separated by sodium dodecyl sulfate-polyacrylamide gel electrophoresis (SDS-PAGE) and transferred onto PVDF membranes. After blocking with 5% skim milk, membranes were incubated overnight with primary antibodies, rabbit anti-Lsm12 monoclonal antibody (Abcam, 1:10000 dilution), or mouse anti-Flag monoclonal antibody (Sigma, 1:500 dilution). Then the membranes were incubated with HRP-conjugated anti-rabbit IgG or anti-mouse IgG secondary antibody for 2 h at room temperature. β-actin or GAPDH was used as an internal control. Bands were detected using the ImageQuant LAS 4000 (GE Healthcare Life Sciences, USA).

### RNA sequencing (RNA-seq) and AS analysis

Total RNA was isolated from Lsm12 knockdown or control SCC-25 cells and then used for whole transcriptome sequencing on an Illumina HiSeq 2500 platform by Novogene (Beijing, China). Differentially expressed mRNAs were screened and analyzed using Disease Ontology database to reveal the association of Lsm12 with various kinds of human diseases. GO and KEGG pathway analysis for DEGs were performed to obtain an overview of biological functions and regulatory mechanism of Lsm12.

Notably, AS changes induced by Lsm12 knockdown were analyzed. First, we selected AS events on the basis of FDR < 0.01. Then we focused on the most significant aberrant events (IncLevelDifference >0.3 or IncLevelDifference < −0.3), which were validated in Lsm12 knockdown or control SCC-25 cells using PCR. PCR products were electrophoresed on 3% agarose gel and sequenced. The AS events verified successfully were further detected in Lsm12 overexpression or control cells using PCR and sequencing.

### Cell proliferation assay

To explore the effect of Lsm12 and USO1 with exon 15 on the proliferation of OSCC cells, real-time monitoring of cell proliferation was performed using Real-Time Cell Analyzer (RTCA, xCELLigence, Roche). Cells were seeded at a density of 2 × 10^3^ cells/well into E-plate 96, which contained a biocompatible microelectrode array. Electrical impedance reflects cell viability.

### Cell cycle analysis

For cell cycle analysis, cells were harvested at 70-80% confluence, then centrifuged and suspended with cold PBS. The cells were fixed in 75% cold ethanol overnight at −20 °C and stained with propidium iodide (PI) buffer (50 μg/mL PI, 50 μg/ml RNaseA, 0.1% Triton) for 30 min at 37 °C followed by analysis using flow cytometry (BD Biosciences). The percentage of cells in different phase was calculated.

### Colony formation assay

Cells were digested and seeded at low density of 1 × 10^3^ cells per well into six-well plates. 15 days for SCC-25 cells and 21 days for CAL 27 cells later, cells were fixed with methanol and stained by crystal violet solution for 10 min. After washing with water, the colonies in each group were examined and counted.

### Migration and invasion assays

Transwell migration and invasion assays were performed using a transwell plate coated with (for invasion) or without (for migration) Matrigel. Briefly, 2.5 × 10^5^ cells were seeded in 100 μl of serum-free medium on the upper chamber and 600 μl of medium containing 40% FBS was added to the lower chamber. SCC-25 and CAL 27 cells were incubated for 24-36 h and 12-16 h at 37 °C with 5% CO_2_, respectively. Cells inside the upper chamber had been wiped away by cotton swabs. Cells that had migrated or invaded though the membrane were fixed with methanol, stained with crystal violet solution and finally photographed under a microscope (Leica, Germany). Five random microscopic fields were counted per well and the mean was determined.

### *In vivo* tumorigenesis assay

4-5 weeks old BALB/C nude mice were obtained from Beijing Vital River Laboratory Animal Technology (Beijing, China). 1.1 × 10^7^ Lsm12 knockdown or control SCC-25 cells were subcutaneously injected into each of five male nude mice. 3.5 × 10^6^ USO1-FL or USO1-DE15 cells were subcutaneously injected into each of six nude mice (3 male and 3 female). *In vivo* solid tumors were dissected and weight. Tumor size was measured with a calliper and the tumor volume was calculated by use of a formula (L × W^2^)/2, where L is the length and W is the width of the tumor. Formalin-fixed, paraffin-embedded tumor specimens were histologically observed by HE staining.

### Statistical analysis

Statistical analysis was performed using SPSS 24 and GraphPad Prism 6 (GraphPad Software, San Diego, CA, USA). Comparisons between the two groups were performed using the unpaired Student’s t-test. Lsm12 IHC scores were compared between OSCC tissues and the paired normal tissues using the paired Student’s t-test. Data are presented as the mean ± SEM. To compare the expression degree of Lsm12 between OSCC and the paired adjacent normal tissues, Spearman’s rho correlation test was used. *P* < 0.05 was considered statistically significant.

## Results

### Progression of OSCC tumors

To explore the biomarker of OSCC, we first established the golden hamster model of OSCC tumorigenesis induced by DMBA, which was confirmed by histopathology (Fig. 1b, Supplementary Fig. S1a). Histopathological images showed that the epithelium in the control group presented keratinized squamous epithelium. Appearance of the buccal mucosa was thin, pink and soft. After DMBA treatment for 4 weeks, hyperplasia could be observed. Buccal mucosa was red and thickened. Mild/moderate dysplasia lesion was evident after 7 weeks of DMBA treatment. The buccal mucosa appeared rough, thickened and lose elasticity. After DMBA treatment for 10 weeks, papilloma with dysplasia could be clearly observed. The papilloma shown in Fig. 1b was accompanied by severe dysplasia. Finally, carcinoma stage was reached after 14 weeks of DMBA treatment. The tumor incidence was 100%.

### Lsm12 expression is upregulated in the model of OSCC tumorigenesis

Next, RNAs isolated from the eighteen frozen specimens including two normal tissues and four tissues per group (hyperplasia, mild/moderate dysplasia, papilloma and squamous cell carcinoma group) were subjected to mRNA microarray assay and 1555 DEGs were identified (fold change≥1.5, *P* < 0.05) and shown in Supplementary Table S1. Microarray analysis indicated that genome-wide expression profiles in the four carcinoma tissues (purple) were similar and much different from profiles of other groups. The genomic expression profiles in two normal tissues (red) were similar (Fig. 1c).

Series test of cluster (STC) algorithm was used to analyze the dynamics of gene expression and explore the differentially expressed mRNA expression profiles. STC analysis showed all DEGs were clustered into 68 profiles, among which 13 profiles containing 665 DEGs exhibited a very significant statistical difference (*P* < 0.001) (Supplementary Fig. S1b). Among 13 very significant profiles, we focused on the upregulated DEGs in the progression of tumorigenesis, especially upregulated at the malignant transformation stage from papilloma to OSCC. After all, important changes of genes expression might be more likely to occur at the malignant transformation stage of papilloma.

Profile #41, #44, #68, #71 showed upregulated trends in the process of OSCC carcinogenesis and from these four profiles we chose 39 genes upregulated by at least 1.5-fold (Supplementary Table S2), among which unknown tumorigenesis-related genes were selected and validated in the animal model using real time PCR. The results of real time PCR analysis showed that Lsm12 expression was significantly upregulated in OSCC tissues compared with papilloma tissues (Fig. 1d), which was consistent with the results of microarray data analysis.

Pathway analysis of DEGs showed some tumor-related signal transduction pathways such as transcriptional misregulation in cancer were involved in OSCC tumorigenesis (Supplementary Fig. S1c).

### Lsm12 expression is remarkably up-regulated in OSCC clinical tissues

To examine whether Lsm12 is also highly expressed in OSCC clinical tissues, we constructed OSCC tissue microarrays composed of 101 cases of OSCC tissues and the paired adjacent normal tissues. The clinical characteristics of these participants are listed in Table 1. Patients below the age of 60 years (73/101, 72.3%) and males (80/101, 79.2%) were the majority. Most of male patients were smokers (65/80, 81.3%).

**Table 1:** Clinical characteristics of the 101 cases of OSCC patients

Variable	Cases, *N* (%)
Gender	
Male	80 (79.2%)
Female	21 (20.8%)
Age	
< 60	73 (72.3%)
> =60	28 (27.7%)
Smoking	
Yes	66 (65.3%)
No	35 (34.7%)
Differentiation	
Well	52 (51.5%)
Moderate	32 (31.7%)
Poor	16 (15.8%)
Undifferentiated	1 (1%)

**Fig. 2:**
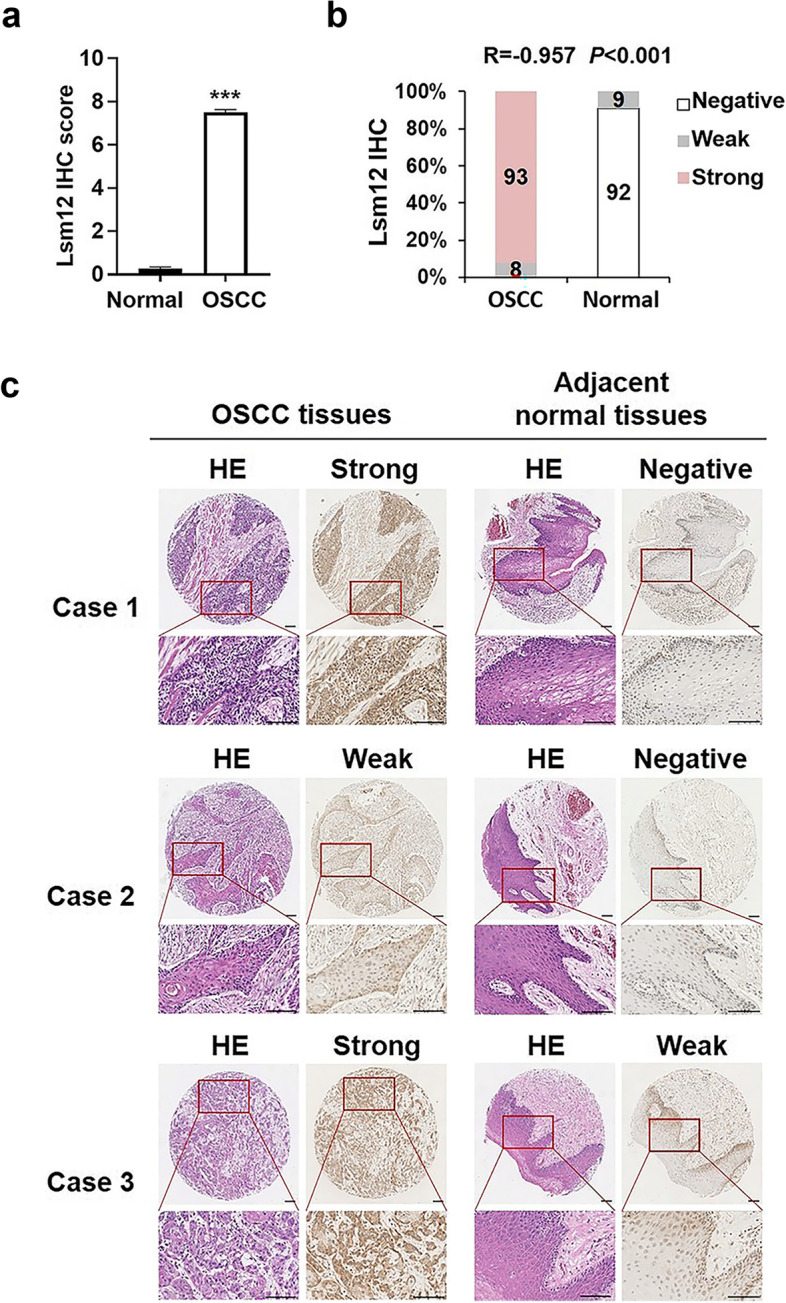
Lsm12 expression is upregulated in OSCC clinical tissues. **a** Statistics analysis of Lsm12 immunohistochemistry scores for the paired samples from 101 OSCC cases. **b** Lsm12 staining was positive in 100% of OSCC tissues and was negative in 91% of the paired normal tissues. Statistics analysis revealed that Lsm12 expression was upregulated significantly in OSCC tissues. **c** IHC staining and HE histopathology images of three representative cases showed Lsm12 expression in OSCC and the paired adjacent normal tissues. Lsm12 expression in the upper spinous and superficial layers was all negative in the paired normal tissues. Bar, 100 μm

The protein levels of Lsm12 in clinical samples were then examined by immunohistochemistry technique. Statistical analysis of Lsm12 immunohistochemistry scores showed that the protein expression of Lsm12 was significantly upregulated in OSCC tissues compared with the paired normal tissues (*P* < 0.001, Fig. 2a). The negative rate of Lsm12 expression in the paired normal tissues was 91.1% (92/101) and only 9 cases were weak positive. Interestingly, weak positive staining was confined to the basal or lower spinous layers so expression of Lsm12 in the upper spinous and superficial layers was all negative in 101 cases of paired normal tissues. In 101 cases of OSCC tissues, the positive rate of Lsm12 expression was 100%. Strong staining spread over the whole OSCC tissues in 93 cases (93/101, 92.1%) and weak staining was in only 8 cases of high differentiated OSCC. IHC staining and HE histopathology images of three representative cases showed higher expression levels of Lsm12 in OSCC tissues than in the paired normal tissues. Collectively, Lsm12 expression is significantly upregulated in OSCC clinical tissues (*P* < 0.001) (Fig. 2b and c).

### The expression levels of Lsm12 are significantly higher in many types of human malignant tumors than in the paired normal tissues

**Fig. 3:**
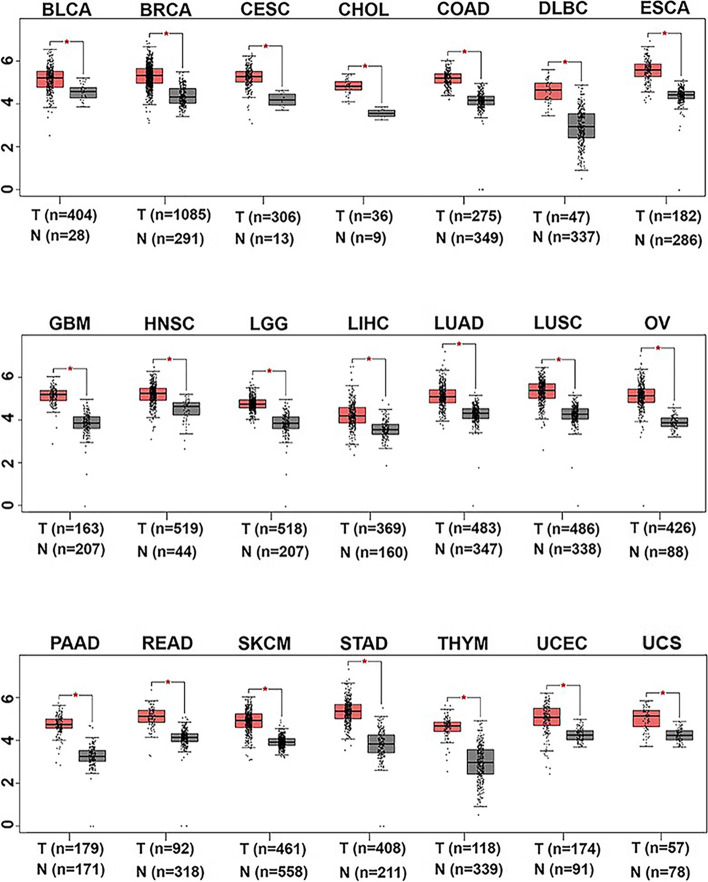
Lsm12 expression is upregulated in many types of malignant tumors. GEPIA database showed that Lsm12 expression was significantly upregulated (fold change ≥1.5) in many types of malignant tumor samples (red box) compared with the paired normal tissues (grey box). Each dot represents expression of one sample. * indicates *P* < 0.05

To investigate the expression of Lsm12 in the other malignant tumors, we used GEPIA database to find out that Lsm12 is also upregulated in many types of malignant tumors compared with the paired normal tissues, such as BRCA (Breast invasive carcinoma), CESC (Cervical squamous cell carcinoma and endocervical adenocarcinoma), CHOL (Cholangio carcinoma), COAD (Colon adenocarcinoma) (Fig. 3), which suggests that Lsm12 is closely associated with the tumorigenesis of human malignant tumors.

### Lsm12 promotes OSCC cell proliferation, motility and tumor formation *in vivo*

To investigate the biological role of Lsm12 in the tumorigenesis, we established the stable OSCC cell lines with Lsm12 overexpression or knockdown. Immunofluorescence images showed that lentivirus containing Lsm12 cDNA or control lentivirus successfully infected CAL 27 and SCC-25 cells (Supplementary Fig. S2a). Real time PCR analysis confirmed that the expression level of Lsm12 was significantly higher in Lsm12-overexpressed SCC-25 and CAL 27 cells than in their respective control cells (Supplementary Fig. S2b). The same results were also obtained by western blotting analysis (Supplementary Fig. S2c and d).

Although SCC-25 cells infected with lentivirus containing Lsm12 shRNA could survive after puromycin selection, these cells grew very poorly while the control cells grew quite well. Real time PCR and western blotting assays showed that expression of Lsm12 was efficiently knocked down in SCC-25 cells (Supplementary Fig. S2b and d). Interestingly, however, CAL 27 cells failed to generate the stable Lsm12 knockdown cell line because almost no cells could survive after they were infected with lentivirus containing Lsm12 shRNA followed by puromycin selection, while the control cells grew very well.

**Fig. 4:**
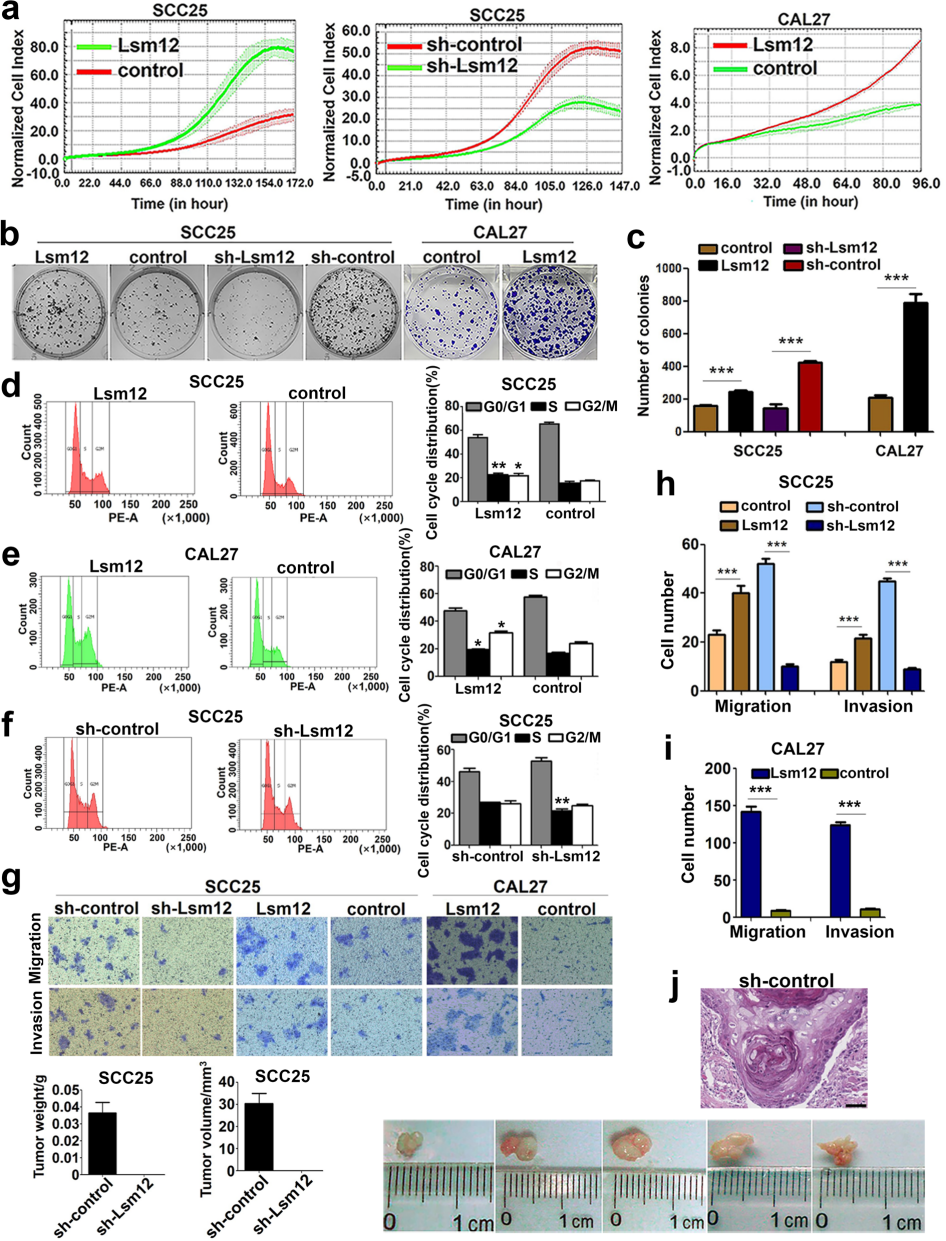
Lsm12 promotes the cell proliferation, mobility and tumorigenicity *in vivo.* **a** Cell growth curve analysis showed that the growth rate of OSCC cells was significantly increased when Lsm12 was overexpressed and decreased upon Lsm12 knockdown. **b, c** Colony formation assay revealed that colony formation ability of OSCC cells was significantly increased when Lsm12 was overexpressed and decreased upon Lsm12 knockdown. **d-f** Cell cycle analysis showed that Lsm12 overexpression increased the proportions of S phase and G_2_/M phase but Lsm12 knockdown decreased the proportion of S phase significantly in OSCC cells. **g-i** Transwell assay showed the effect of Lsm12 on the migration and invasion abilities of OSCC cells. **j** The results of *in vivo* tumorigenicity assay showed that no tumor formation was observed in five mice of Lsm12 knockdown group and the tumors were formed in five mice of the control group. Histopathological examination of the excised tumors showed squamous cell carcinoma. Bar, 50 μm. *, ** and *** indicate *P* < 0.05, *P* < 0.01 and *P* < 0.001, respectively

Functionally, the cell growth curve measured by exCELLigence RTCA MP systems showed that overexpression of Lsm12 greatly increased the growth rates of both SCC-25 and CAL 27 cells. On the other hand, the growth rate of SCC-25 cells was decreased notably upon Lsm12 knockdown (Fig. 4a). Consistently, the ability of colony formation was significantly enhanced in Lsm12 overexpression SCC-25 and CAL 27 cells but was markedly decreased in Lsm12 knockdown SCC-25 cells, as compared to their respective control cells (Fig. 4b and c). Furthermore, cell cycle analysis showed that the proportions of both S phase and G2/M phase were all significantly increased in Lsm12 overexpression SCC-25 and CAL 27 cells but the proportion of S phase was decreased in Lsm12 knockdown SCC-25 cells, as compared with their respective control cells (Fig. 4d-f).

The effect of Lsm12 on the cell motility was detected by Transwell migration and Matrigel invasion assays and the results showed that overexpression of Lsm12 obviously enhanced the migration and invasion abilities of SCC-25 and CAL 27 cells, while knockdown of Lsm12 remarkably inhibited the migration and invasion abilities of SCC-25 cells (*P* < 0.001, Fig. 4g-i).

To investigate the function of Lsm12 on the tumor formation *in vivo*, Lsm12 knockdown SCC-25 cells (1.1 × 10^7^ cells per mouse) or control cells were injected into the nude mice. The results showed that the tumors were formed in all five mice of control group, but no tumor was observed in five mice of Lsm12 knockdown group, which suggested that depletion of Lsm12 could significantly inhibit the tumor formation of OSCC cells *in vivo*. Histopathological examination showed that those tumors were squamous cell carcinoma (Fig. 4j).

### Lsm12 regulates the expression and alternative splicing of tumor-related genes

**Fig. 5:**
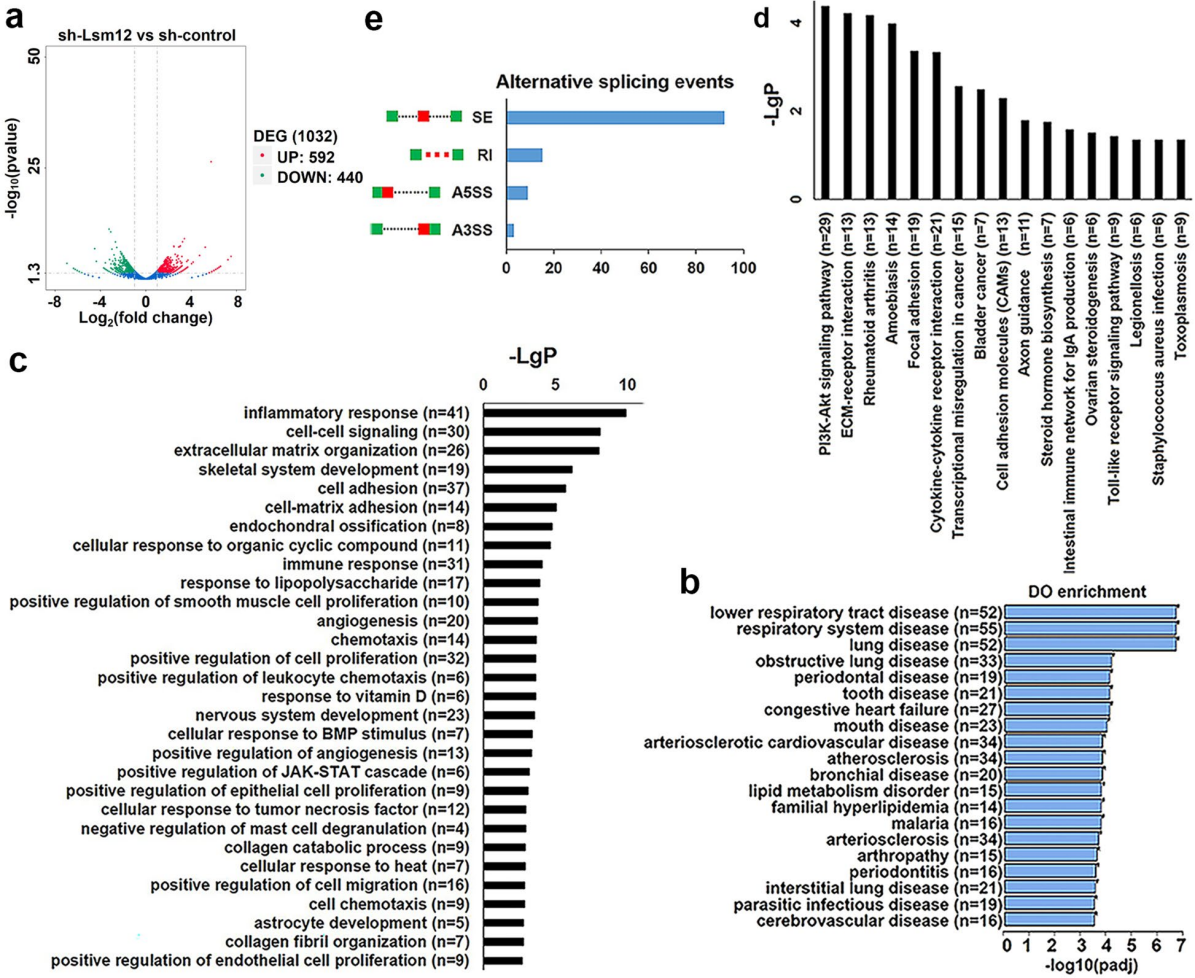
Lsm12 regulates the expression and alternative splicing of genes. **a** Volcano plot showed 1032 DEGs including 592 upregulated and 440 downregulated genes induced by Lsm12 knockdown. The red dots represent the upregulated genes and the green dots represent the downregulated genes (≥2-fold, *P* < 0.05). **b** The top 20 significantly enriched DO categories include some oral and respiratory diseases. **c** The top 30 enriched GO items such as positive regulation of cell proliferation, positive regulation of angiogenesis, and immune response. **d** The enriched 17 pathways such as transcriptional misregulation in cancer. **e** Lsm12 knockdown induced alternative splicing events including SE, RI, A5SS and A3SS

To explore the mechanism of Lsm12 involved in OSCC tumorigenesis, the whole transcriptomes in Lsm12 knockdown SCC-25 cells and the control cells were analyzed through high-throughput RNA sequencing. 1032 DEGs (≥2-fold, *P* < 0.05) were identified, including 592 upregulated and 440 downregulated genes, in Lsm12 knockdown SCC-25 cells (Fig. 5a, Supplementary Table S3). The heatmap of top 20 upregulated/downregulated genes induced by Lsm12 knockdown was shown in Supplementary Fig. S3a.

To explore the association of Lsm12 with human diseases, Disease Ontology (DO) analysis for DEGs was performed and the results showed that Lsm12 were associated with multiple human diseases, such as oral diseases and respiratory diseases. DO items on oral diseases included mouth disease, periodontal disease, tooth disease and periodontitis. DO items on respiratory diseases were lower respiratory tract disease, respiratory system diseases, lung disease, obstructive lung disease, bronchial disease and interstitial lung disease (Fig. 5b). GO analysis and KEGG pathway analysis for DEGs revealed that tumorigenesis-related biological processes, such as immune response, positive regulation of cell proliferation, positive regulation of angiogenesis, cellular response to tumor necrosis factor and positive regulation of migration, and pathways such as transcriptional misregulation in cancer were involved (Fig. 5c and d).

Moreover, Lsm12 knockdown could also cause alternative splicing changes of genes, including SE (skipped exon), RI (retained intron), A5SS (alternative 5′ splice site) and A3SS (alternative 3′ splice site) events (Fig. 5e, Supplementary Table S4).

### Lsm12 regulates alternative splicing of USOI

**Fig. 6:**
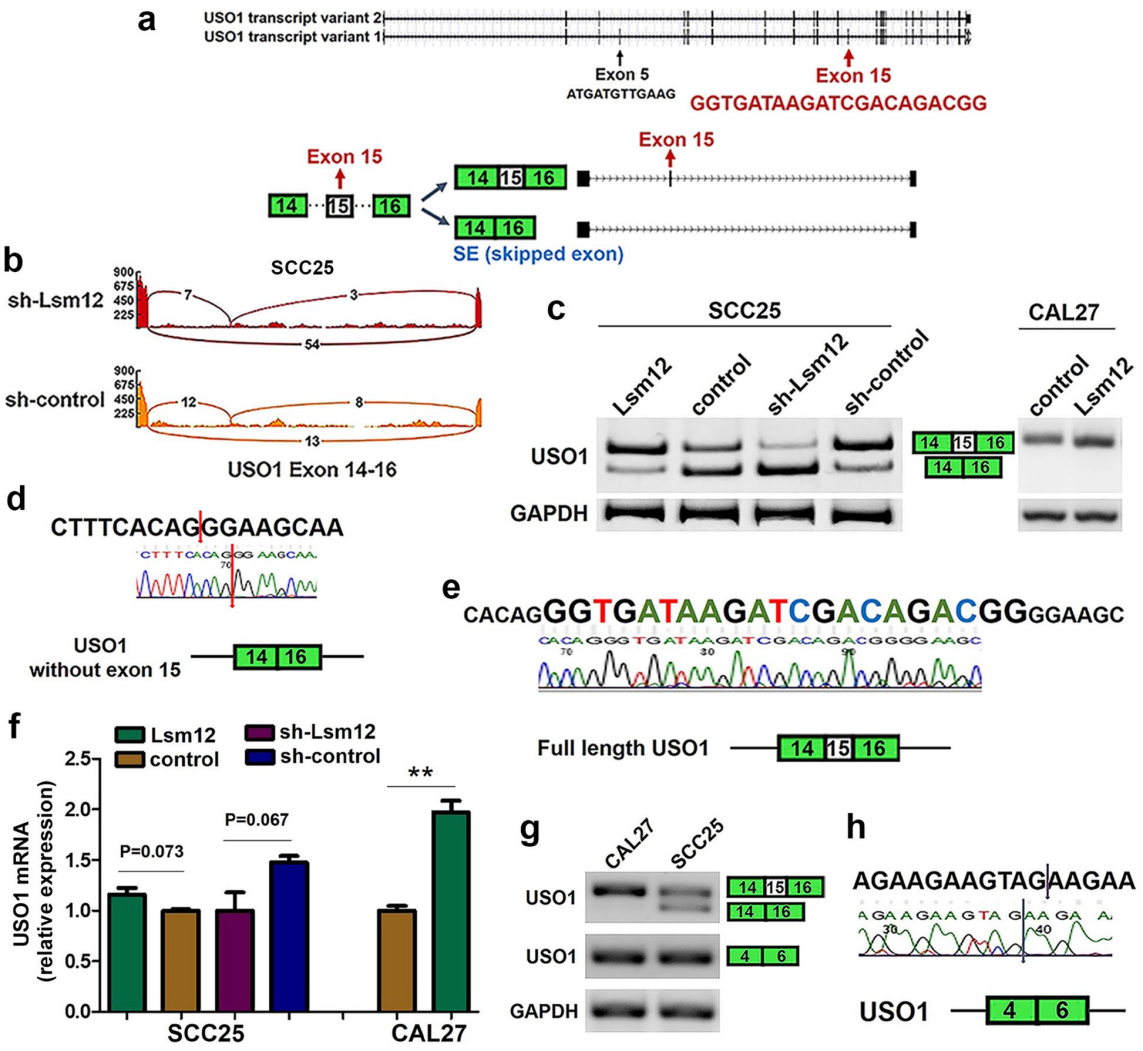
Lsm12 regulates alternative splicing of USO1. **a** USO1 gene has two transcript variants according to UCSC database. Alternative splicing of USO1 exon 15 is shown. **b** Whole transcriptome sequencing analysis revealed that Lsm12 knockdown induced USO1 exon 15 skipping. **c-f** The results of PCR assay and sequencing showed that Lsm12 regulated the alternative splicing of USO1 exon 15 in SCC-25 cells and modulated the expression level of USO1 in CAL 27 cells. Red arrows indicate exon 15 skipping. **g, h** PCR assay and sequencing showed that there were two USO1 transcript variants with or without exon 15 in SCC-25 cells, but only one USO1 transcript variant with exon 15 was observed in CAL 27 cells. USO1 exon 5 was found constitutively to be skipped in both SCC-25 and CAL 27 cells. Blue arrows indicate Exon 5 skipping

According to UCSC databases, USO1 has two transcript variants, variant 1 (NM_001290049) and variant 2 (NM_003715). Transcript variant 1 contains 26 exons, and variant 2 contains 24 exons which lacks exon 5 and exon 15 of variant 1 (Fig. 6a). Whole transcriptome sequencing analysis revealed that Lsm12 knockdown induced USO1 exon 15 skipping (Fig. 6b, Supplementary Table S4). Because IncLevelDifference is −0.451, alternative splicing of USO1 exon 15 was selected to be validated in Lsm12 knockdown cells and control cells by PCR assay and sequencing of amplified products. The results of PCR assay showed that knockdown of Lsm12 notably increased the level of USO1 without exon 15, but decreased the level of USO1 containing exon 15 in SCC-25 cells. Conversely, Lsm12 overexpression markedly increased the level of USO1 containing exon 15 and obviously decreased the level of exon 15-deleted USO1 (Fig. 6c). These results were further confirmed by sequencing of PCR products on agarose gel (Fig. 6d, e). Taken together, our results indicated that Lsm12 overexpression resulted in an obvious inclusion of USO1 exon 15 and Lsm12 knockdown caused a marked skipping of USO1 exon 15 in SCC-25 cells. In a word, Lsm12 can regulate the alternative splicing of USO1 exon 15.

Intriguingly, in Lsm12 overexpression CAL 27 cells and control CAL 27 cells, only one band for USO1 containing exon 15 could be observed (Fig. 6c). The results of real time PCR revealed that expression level of USO1 was significantly higher in Lsm12 overexpression CAL 27 cells than in the control cells (*P* < 0.01, Fig. 6f). There was no significant change in the expression level of USO1 in SCC-25 cells as Lsm12 was overexpressed or knocked down (Fig. 6f).

The sequence of USO1 exon 15 is GGTGATAAGATCGACAGACGG (21 bp) and sequence of exon 5 is ATGATGTTGAAG (12 bp) (Fig. 6a). In the parent SCC-25 cells, there were two USO1 transcript variants, with and without exon 15. In the parent CAL 27 cells, only one USO1 transcript variant with exon 15 was observed. In both SCC-25 and CAL 27 cells, exon 5 was all found constitutively to be skipped (Fig. 6g and h). Therefore, a novel USO1 transcript variant with exon 15 and without exon 5 was discovered in OSCC cells.

### USO1 containing exon 15 promotes the cell proliferation, motility and tumor formation *in vivo*

**Fig. 7:**
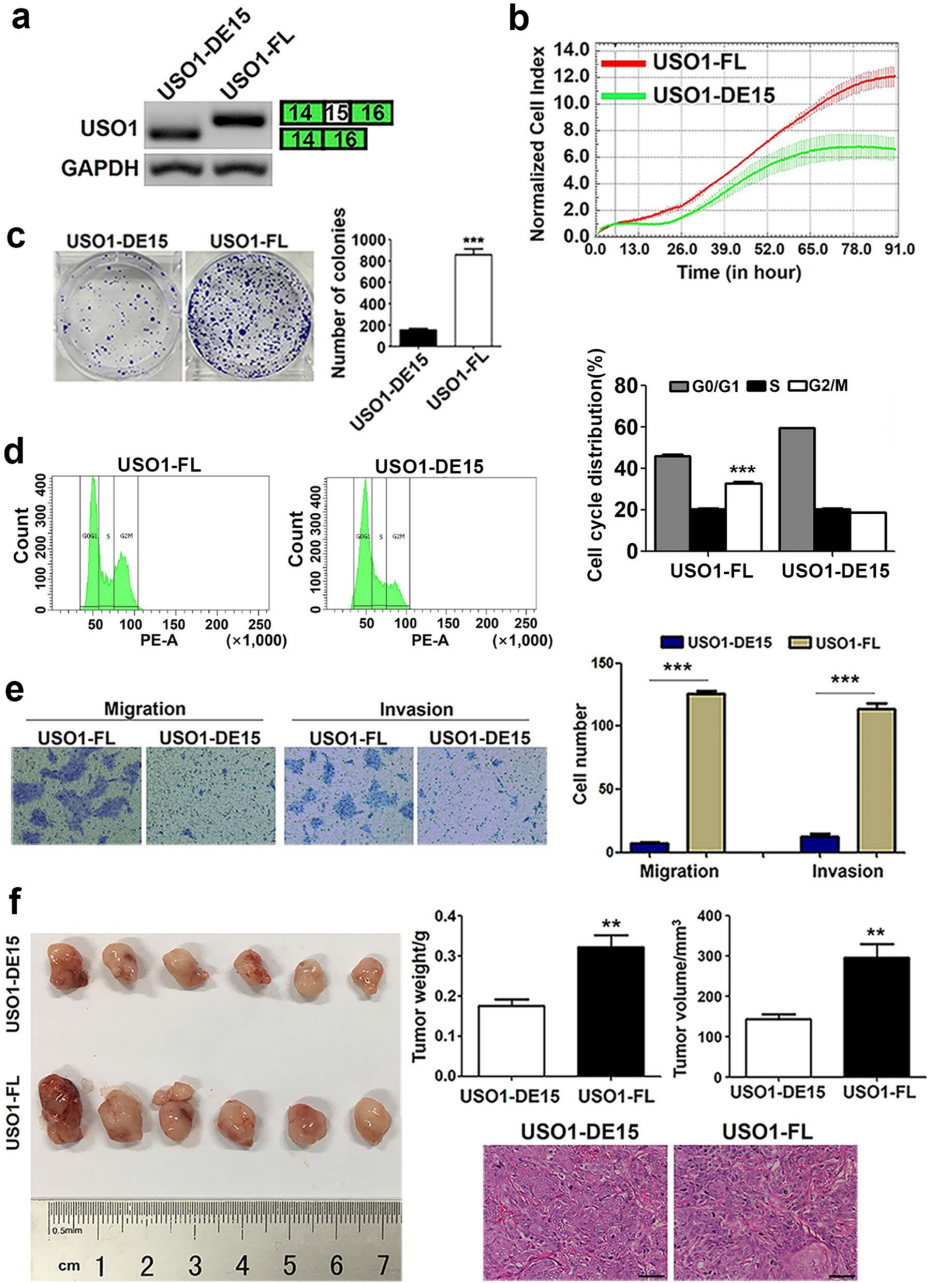
USO1 containing exon 15 promotes cell proliferation, mobility and tumorigenicity *in vivo.* **a** The results of PCR assay and sequencing showed that the stable cell lines overexpressing USO1 with or without exon 15 were constructed in CAL 27 cells. **b**-**e** Overexpression of USO1 containing exon 15 significantly promoted the cell proliferation, increased the proportion of G_2_/M phase, and enhanced the colony formation, cell migration and invasion abilities of OSCC cells. **f** 3.5 × 10^6^ USO1-FL or USO1-DE cells were subcutaneously injected into each of six nude mice. Excised tumors were shown. Tumor volume and weight were increased remarkably in the group of USO1 with exon 15, compared with those in the group of USO1 without exon 15. Histopathological examination of the tumor specimens from these two groups showed squamous cell carcinoma. Bar, 50 μm

Immunofluorescent images showed that the lentivirus containing full length USO1 or exon 15-deleted USO1 infected CAL 27 cells successfully (Supplementary Fig. S3b). The results of PCR assay and sequencing of the PCR products verified that only one band of exon 15-retained USO1 or exon 15-deleted USO1 could be observed in USO1-FL or USO1-DE15 cells, respectively (Fig. 7a, Supplementary Fig. S3c).

The effects of USO1 with or without exon 15 on the tumorigenesis were explored. Cell growth curve showed that the growth rate of USO1-FL cells was significantly increased compared with USO1-DE15 cells (Fig. 7b), suggesting that USO1 exon 15 is involved in the OSCC cell growth. Consistently, the results of colony formation assay revealed that the ability of colony formation was significantly enhanced in USO1-FL cells, compared with that in USO1-DE15 cells (*P* < 0.001, Fig. 7c). To further confirm the importance of USO1 exon 15 in the proliferation of OSCC cells, cell cycle analysis was performed and the results revealed that the percentage of G_2_/M phase was significantly increased in USO1-FL cells as compared with that in USO1-DE15 cells (*P* < 0.001, Fig. 7d). To detect the effects of USO1 containing exon 15 on the cell motility, we performed Transwell migration and invasion assays. As shown in Fig. 7e, more migrated and invaded cells were observed in USO1-FL cells than in USO1-DE15 cells (*P* < 0.001).

To investigate the importance of USO1 exon 15 in the tumor formation *in vivo*, tumorigenesis assay in nude mice was performed. The results revealed that the tumor volume and tumor weight were remarkably decreased in the group of exon 15-deleted USO1, compared with those in the group of full length USO1 (*P* < 0.01). Histopathological examination of the tumor specimens from the two groups showed squamous cell carcinoma (Fig. 7f).

## Discussion

In this study, we established the carcinogenesis model of OSCC in golden hamsters and then analyzed differentially expressed mRNAs among normal, hyperplasia, mild/moderate dysplasia, papilloma and squamous cell carcinoma (SCC) tissues in the model, which showed the dynamic changes of genome expression in the tumorigenesis. Interestingly, we identified a novel RNA-splicing related gene Lsm12 as an important regulator involved in OSCC tumorigenesis and made the first demonstration that Lsm12 is a tumorigenesis-associated gene.

However, the molecular function of Lsm12 remained largely unclear although Lsm12 expression level is also significantly higher in many other types of human tumors besides OSCC. In our immunohistochemistry analysis, Lsm12 protein expression was found significantly upregulated in OSCC tumors compared with their paired normal tissues. Lsm12 staining spread over the whole cancer tissues while in 91% of normal tissues, Lsm12 staining was negative. Interestingly, even in a few normal tissues with weak Lsm12 staining, weak staining was only confined to the base layer or lower spinous layer. The distribution characteristics of Lsm12 protein suggested that it might have important clinical implications. It might be used as a strong biomarker for large-scale screening of malignant transformation in oral mucosa lesions by oral scraping or for determining the scope of surgical resection in the therapy of OSCC.

To date, the “gold standard” for OSCC diagnosis is still a clinical oral examination integrated by a histopathological examination on the biopsy of suspicious lesions [33]. However, cancer research focuses on finding less invasive and more effective methods to make an early diagnosis, monitor its evolution and therapeutic response [34–43]. For example, cervical scraping has been commonly used to screen for cervical cancer in clinical examination [36, 37]. Liquid biopsy is also a less invasive method to detect the diagnostic biomarkers in body fluids such as urine or blood, but for OSCC and many malignant tumors, markers could be identified in the body fluid at the advanced stage, not early stage. For example, CrisafulliG et al. isolated DNA from plasma and urine of metastatic colorectal cancer patients to analyze the tumor-related genetic information by whole exome sequencing [39]. Although salivary biomarkers for oral cancer, such as defensin-1, P53, TNF-α and Cyfra 21-1, have been identified, the reliability and validation of salivary biomarkers for clinical applications need further researches [43].

Therefore, oral scraping might be a good method to screen for early malignant transformation in oral mucosa lesions. By scraping from the oral lesions, we could obtain the upper spinous cells and superficial epithelial cells in which the degree of Lsm12 staining by immunohistochemistry could help us diagnose OSCC. Abnormal lesions in the oral mucosa are easy to find and observe and thus oral scraping is a quick and easy operation without bleeding, so it could effectively avoid triggering hematogenous metastasis of malignant cells. In addition, in the therapy for OSCC, the level of Lsm12 expression might provide a basis for surgeons to determine the scope of surgical resection. After all, margin resection status is a major risk factor for the local recurrence of cancers.

Moreover, we investigated the biological function of Lsm12 and demonstrated that Lsm12 overexpression significantly promoted the cell growth, colony formation, cell invasion and migration, while Lsm12 knockdown obviously inhibited these phenotypes, which suggested that upregulation of Lsm12 is an important mechanism of OSCC tumorigenesis.

Interestingly, during establishment of stable Lsm12 knockdown cell line, we observed that SCC-25 cells infected with lentivirus containing Lsm12 shRNA grew very poorly, and CAL 27 cells even failed to generate the stable Lsm12 knockdown cell line because almost no cells could survive after they were infected with this lentivirus, indicating that Lsm12 may be important for the cell survival. In vivo tumor formation assay showed that no tumor was formed when Lsm12 was knocked down, which might be due to the cell death induced by Lsm12 knockdown.

Lsm12 belongs to Sm family involved in RNA splicing. As expected for Lsm12’s role, Lsm12 exactly played a key role in RNA splicing. Whole transcriptome sequencing showed that Lsm12 knockdown could induce alternative splicing changes of many genes. Aberrant RNA splicing is recognized to contribute to carcinogenesis. In the present study, we found that Lsm12 overexpression enhanced the inclusion of USO1 exon 15 and Lsm12 knockdown promoted the deletion of USO1 exon 15 in OSCC cells. Taken together, Lsm12 could regulated the alternative splicing of USO1 exon 15.

UCSC database showed that USO1 has two transcript variants shown in Fig. 6a. In our study, a novel USO1 transcript variant containing exon 15 but lacking exon 5 was found in OSCC cells. Some studies have reported that USO1 is involved in the tumorigenesis of certain human malignant tumors [28–31]. For example, USO1 knockdown inhibits cell proliferation and induces cell apoptosis in multiple myeloma cells [28]. Downregulation of USO1 suppresses cell proliferation and migration, results in early apoptosis and reduces proportion of G_2_-M phase in human colon cancer cells [29].

In our study, we discovered that overexpression of USO1 containing exon 15 significantly promoted cell proliferation, cell migration and invasion, increased the portion of cells in G_2_-M phase and enhanced tumorigenicity *in vivo*, compared with exon 15-deleted USO1, suggesting that USO1 containing exon 15 played a key role in OSCC tumorigenesis. Likely, overexpression of USO1 containing exon 15 is an important mechanism for USO1 gene involved in tumorigenesis.

## Conclusions

In summary, we identify Lsm12 as a novel tumorigenesis-associated gene involved in OSCC tumorigenesis. Lsm12 is significantly upregulated in OSCC tissues as compared to the paired normal tissues. Overexpression of Lsm12 promote the malignant characters of OSCC cells. Lsm12 is able to modulate the alternative splicing of genes, such as USO1. Lsm12 favors the production of exon 15-retained USO1, which is an important mechanism of USO1 involved in the tumorigenesis. Our findings thus provide that Lsm12 could be a potent biomarker of OSCC tumorigenesis and a potential target for the therapy of OSCC.

## Supplementary Information

**Additional file 1: Fig. S1.** Gene expression profiles and pathway analysis of DEGs identified in the model of OSCC tumorigenesis. a The resected buccal mucosa in the animal model of OSCC tumorigenesis. b 13 very significant gene expression profiles contain 665 DEGs with *p* < 0.001, among which Profile #44, #71, #68 and #41 in the blue boxes showed upregulated trends in the process of carcinogenesis. Each profile contains a group of genes with a similar expression pattern. The horizontal and vertical axes represent time points and normalized gene expression levels, respectively. c Pathway analysis showed 21 enriched pathways such as transcriptional misregulation in cancer and pathways in cancer. **Fig. S2.** Lsm12 overexpression or knockdown stable cell lines were constructed. a Immunofluorescence images showed that lentivirus containing Lsm12 cDNA infected SCC-25 and CAL 27 cells. b-d The results of real time PCR and western blotting assay confirmed that Lsm12 overexpression or knockdown stable cell lines were established. **Fig. S3.** DEGs induced by Lsm12 knockdown and the stable cell lines overexpressing USO1 with or without exon 15. a The heatmap of top 20 upregulated/downregulated genes induced by Lsm12 knockdown. b Immunofluorescence images showed that lentivirus carrying full length USO1 or exon 15-deleted USO1 infected CAL 27 cells successfully. c The sequencing of PCR products confirmed the overexpression of full length USO1 in USO1-FL cells and overexpression of exon 15-deleted USO1 in USO1-DE15 cells. Red arrows indicate Exon 15 skipping.

**Additional file 2: Table S1.** Differentially expressed genes identified in the animal model of OSCC tumorigenesis.

**Additional file 3: Table S2.** Upregulated genes in the animal model of OSCC tumorigenesis.

**Additional file 4: Table S3.** Differentially expressed genes induced by Lsm12 knockdown.

**Additional file 5: Table S4.** Alternative splicing of genes induced by Lsm12 knockdown.

## Data Availability

All the data obtained and/or analyzed during the current study were available from the corresponding authors on reasonable request.

## Abbreviations

BLCA: Bladder urothelial carcinoma
BRCA: Breast invasive carcinoma
CESC: Cervical squamous cell carcinoma and endocervical adenocarcinoma
CHOL: Cholangio carcinoma
COAD: Colon adenocarcinoma
DLBC: Lymphoid neoplasm diffuse large B-cell lymphoma
ESCA: Esophageal carcinoma
GBM: Glioblastoma multiforme
HNSC: Head and neck squamous cell carcinoma
LGG: Brain lower grade glioma
LIHC: Liver hepatocellular carcinoma
LUAD: Lung adenocarcinoma
LUSC: Lung squamous cell carcinoma
OV: Ovarian serous cystadenocarcinoma
PAAD: Pancreatic adenocarcinoma
READ: Rectum adenocarcinoma
SKCM: Skin cutaneous melanoma
STAD: Stomach adenocarcinoma
THYM: Thymoma
UCEC: Uterine corpus endometrial carcinoma
UCS: Uterine carcinosarcoma
