# Supplementary material for: Identification of RNA-splicing factor Lsm12 as a novel tumor-associated gene and a potent biomarker in Oral Squamous Cell Carcinoma (OSCC)
Source: J Exp Clin Cancer Res. 2022 Apr 21;41:150. doi: 10.1186/s13046-022-02355-9 (PMC9027881; doi:10.1186/s13046-022-02355-9)
Supplement: Supplementary file 1 — Additional file 1: Fig. S1. Gene expression profiles and pathway analysis of DEGs identified in the model of OSCC tumorigenesis. a The resected buccal mucosa in the animal model of OSCC tumorigenesis. b 13 very significant gene expression profiles contain 665 DEGs with p < 0.001, among which Profile #44, #71, #68 and #41 in the blue boxes showed upregulated trends in the process of carcinogenesis. Each profile contains a group of genes with a similar expression pattern. The horizontal and vertical axes represent time points and normalized gene expression levels, respectively. c Pathway analysis showed 21 enriched pathways such as transcriptional misregulation in cancer and pathways in cancer. Fig. S2. Lsm12 overexpression or knockdown stable cell lines were constructed. a Immunofluorescence images showed that lentivirus containing Lsm12 cDNA infected SCC-25 and CAL 27 cells. b-d The results of real time PCR and western blotting assay confirmed that Lsm12 overexpression or knockdown stable cell lines were established. Fig. S3. DEGs induced by Lsm12 knockdown and the stable cell lines overexpressing USO1 with or without exon 15. a The heatmap of top 20 upregulated/downregulated genes induced by Lsm12 knockdown. b Immunofluorescence images showed that lentivirus carrying full length USO1 or exon 15-deleted USO1 infected CAL 27 cells successfully. c The sequencing of PCR products confirmed the overexpression of full length USO1 in USO1-FL cells and overexpression of exon 15-deleted USO1 in USO1-DE15 cells. Red arrows indicate Exon 15 skipping. [file 13046_2022_2355_MOESM1_ESM.pdf]

Supplementary Figures

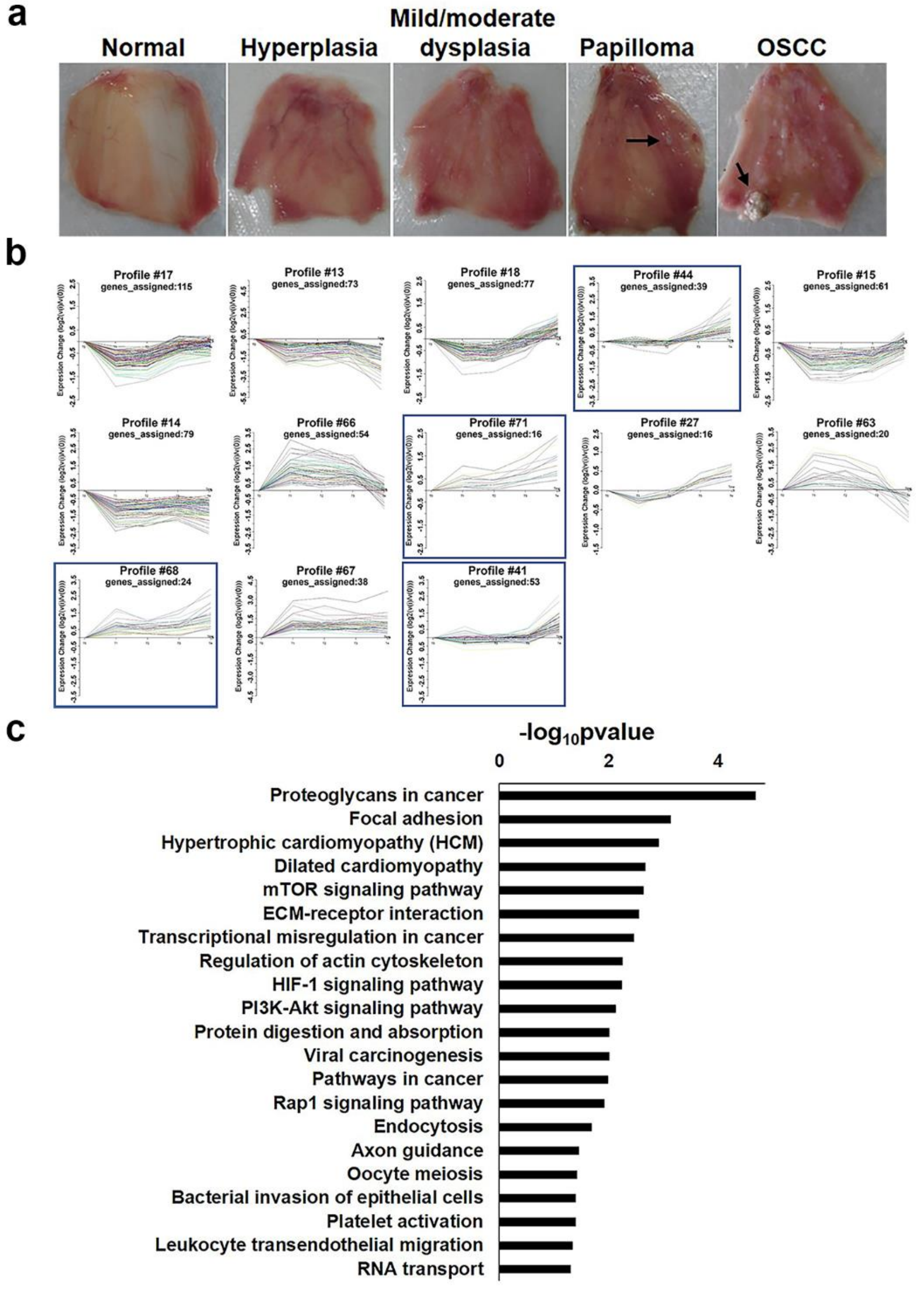

Fig. S1 Gene expression profiles and pathway analysis of DEGs identified in the model of OSCC tumorigenesis

a The resected buccal mucosa in the animal model of OSCC tumorigenesis. **b** 13 very significant gene expression profiles contain 665 DEGs with p<0.001, among which

Profile #44, #71, #68 and #41 in the blue boxes showed upregulated trends in the process of carcinogenesis. Each profile contains a group of genes with a similar expression pattern. The horizontal and vertical axes represent time points and normalized gene expression levels, respectively. **c** Pathway analysis showed 21 enriched pathways such as transcriptional misregulation in cancer and pathways in cancer.

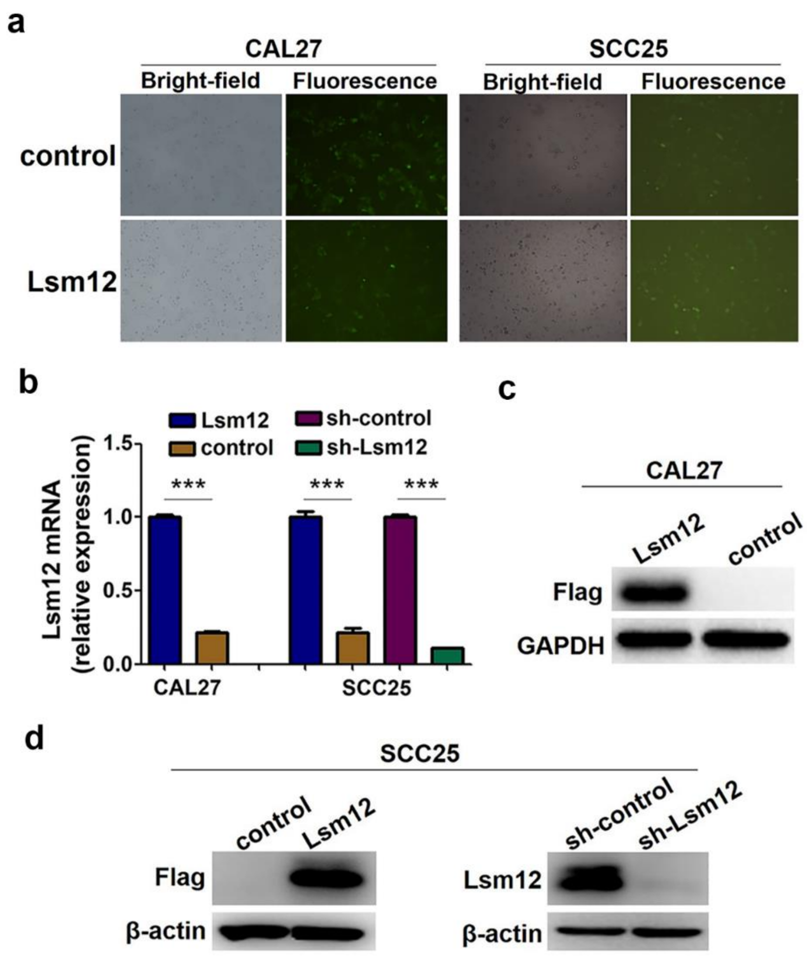

Fig. S2 Lsm12 overexpression or knockdown stable cell lines were constructed

a Immunofluorescence images showed that lentivirus containing Lsm12 cDNA infected SCC-25 and CAL 27 cells. **b-d** The results of real time PCR and western blotting assay confirmed that Lsm12 overexpression or knockdown stable cell lines were established.

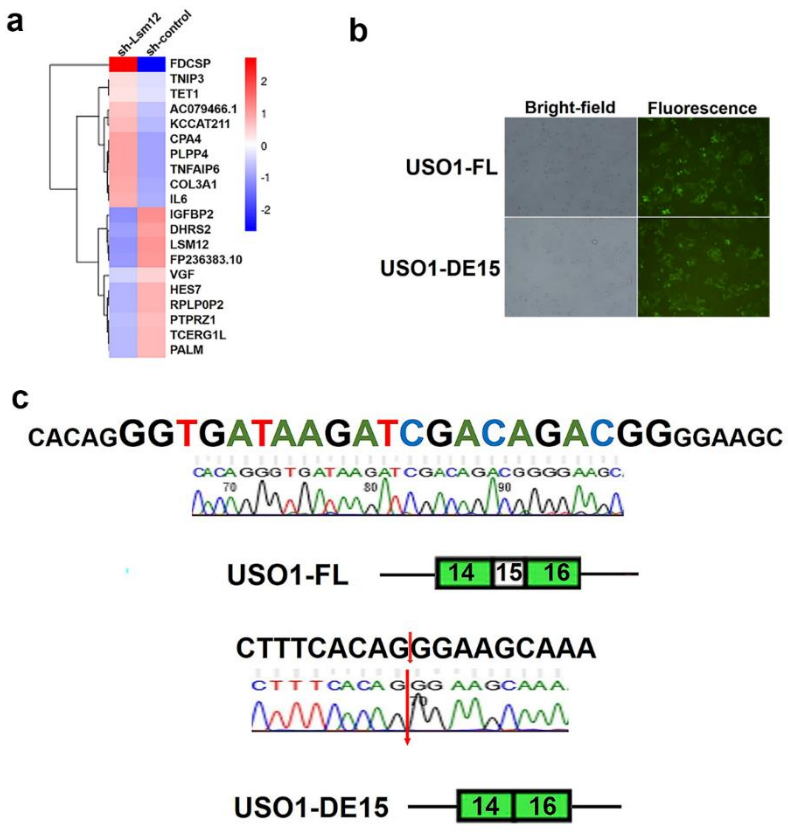

Fig. S3 DEGs induced by Lsm12 knockdown and the stable cell lines overexpressing USO1 with or without exon 15

a The heatmap of top 20 upregulated/downregulated genes induced by Lsm12 knockdown. **b** Immunofluorescence images showed that lentivirus carrying full length USO1 or exon 15-deleted USO1 infected CAL 27 cells successfully. **c** The sequencing of PCR products confirmed the overexpression of full length USO1 in USO1-FL cells and overexpression of exon 15-deleted USO1 in USO1-DE15 cells. Red arrows indicate Exon 15 skipping.
